# Improved workflow for quantification of left ventricular volumes and mass using free-breathing motion corrected cine imaging

**DOI:** 10.1186/s12968-016-0231-8

**Published:** 2016-02-25

**Authors:** Russell Cross, Laura Olivieri, Kendall O’Brien, Peter Kellman, Hui Xue, Michael Hansen

**Affiliations:** Division of Cardiology, Children’s National Health System, 111 Michigan Avenue, NW, Washington, DC, 20010 USA; National Heart, Lung, and Blood Institute, National Institutes of Health, Bethesda, MD USA

**Keywords:** Retrospective reconstruction, Cardiac function, Cardiac volume, Motion correction, Real-time imaging, Cardiovascular MR, Breath-held, Free-breathing, Cine, Reconstruction time

## Abstract

**Background:**

Traditional cine imaging for cardiac functional assessment requires breath-holding, which can be problematic in some situations. Free-breathing techniques have relied on multiple averages or real-time imaging, producing images that can be spatially and/or temporally blurred. To overcome this, methods have been developed to acquire real-time images over multiple cardiac cycles, which are subsequently motion corrected and reformatted to yield a single image series displaying one cardiac cycle with high temporal and spatial resolution. Application of these algorithms has required significant additional reconstruction time. The use of distributed computing was recently proposed as a way to improve clinical workflow with such algorithms. In this study, we have deployed a distributed computing version of motion corrected re-binning reconstruction for free-breathing evaluation of cardiac function.

**Methods:**

Twenty five patients and 25 volunteers underwent cardiovascular magnetic resonance (CMR) for evaluation of left ventricular end-systolic volume (ESV), end-diastolic volume (EDV), and end-diastolic mass. Measurements using motion corrected re-binning were compared to those using breath-held SSFP and to free-breathing SSFP with multiple averages, and were performed by two independent observers. Pearson correlation coefficients and Bland-Altman plots tested agreement across techniques. Concordance correlation coefficient and Bland-Altman analysis tested inter-observer variability. Total scan plus reconstruction times were tested for significant differences using paired t-test.

**Results:**

Measured volumes and mass obtained by motion corrected re-binning and by averaged free-breathing SSFP compared favorably to those obtained by breath-held SSFP (*r* = 0.9863/0.9813 for EDV, 0.9550/0.9685 for ESV, 0.9952/0.9771 for mass). Inter-observer variability was good with concordance correlation coefficients between observers across all acquisition types suggesting substantial agreement. Both motion corrected re-binning and averaged free-breathing SSFP acquisition and reconstruction times were shorter than breath-held SSFP techniques (*p* < 0.0001). On average, motion corrected re-binning required 3 min less than breath-held SSFP imaging, a 37 % reduction in acquisition and reconstruction time.

**Conclusions:**

The motion corrected re-binning image reconstruction technique provides robust cardiac imaging that can be used for quantification that compares favorably to breath-held SSFP as well as multiple average free-breathing SSFP, but can be obtained in a fraction of the time when using cloud-based distributed computing reconstruction.

## Background

Cardiovascular magnetic resonance (CMR) is widely used for evaluation of ventricular anatomy and function in both adult and pediatric cardiology [1, 2] and is considered the gold-standard for measurement of ventricular volume and function in the resting state [3–6]. Traditionally, standard cine CMR for ventricular volume and functional analysis has utilized balanced steady-state free-precession (SSFP) techniques that use acquisition of data over several heartbeats with k-space segmentation, requiring the patient to maintain breath-holds in order to eliminate respiratory motion artifact [7, 8]. Typically, one to two image slices can be obtained per breath-hold with this approach, requiring several repetitive breath-holds to cover the entire heart. From a clinical perspective, it is sometimes necessary to perform imaging in free-breathing patients, such as young children or others unable to conform to breath-hold commands, in which case multiple signal averaging techniques can be employed [2]. The accuracy and reproducibility of volume and function quantification in free-breathing patients is sometimes questioned due to the image blurring that can result from averaging multiple respiratory states. Additionally, standard k-space segmented SSFP imaging techniques are intolerant of patient motion and arrhythmia, and can take a significant time to acquire (on the order of 12 to 15 s per breath-hold), which can limit its usefulness in sicker patients or young children.

Consequent to the limitations of standard SSFP cine imaging, various alternative techniques have been developed to circumvent the breath-hold requirement and to minimize image acquisition time. One example is real-time cine, which uses rapid single-shot image acquisitions that can be performed without breath-holds or ECG triggering [9–11]. Because of the speed at which the images are obtained, the technique is less sensitive to poor breath-holding performance and arrhythmia, but images are of inferior quality as a result of limitations in spatial and temporal resolution [12]. To overcome the image quality limitations associated with real-time imaging, it has been proposed that a series of real-time images acquired over multiple cardiac cycles can be combined using non-rigid motion correction to yield a high temporal and spatial resolution image series covering the entire cardiac cycle. Initially, it was shown by Kellman et al. [13] that real-time images from multiple cardiac cycles can be motion corrected and averaged to recover signal-to-noise losses associated with high parallel imaging factors. This technique was subsequently refined to include a k-space re-binning scheme that yielded improvements in both signal-to-noise and temporal resolution compared to real-time imaging [14]. In this manuscript we will refer to this type of approach as motion corrected re-binning reconstruction. It has also been shown that such an approach is compatible with non-Cartesian imaging [15]. The extension to arbitrary k-space sampling is achieved by including the motion correction step in the encoding equation and including this constraint in an iterative reconstruction [16]. As these methods have been refined, the amount of required real-time data has decreased from about 60 s of real-time data per slice in the originally proposed method to about 16–20 s in a more recent implementation [12], which included a non-linear reconstruction step to recover large areas of missing data in k-space. So far, this technique has only been evaluated in a limited number (*N* = 15) of volunteers.

With each new layer of sophistication in the reconstruction algorithms, the reconstruction time has increased. In spite of great attention to numerical efficiency of the reconstruction software and the use of high performance reconstruction engines such as the Gadgetron [17], reconstruction times on the order of one minute per slice have been reported [12]. When considering that full cardiac coverage for ventricular function would require 9–12 slices, it means that the data for cardiac function can be acquired in approximately 3 min but the subsequent reconstruction debt would be upwards of 15 min rendering the techniques less than practical for clinical use. However, the use of distributed computing was recently proposed as a way to improve clinical workflow when using sophisticated reconstruction algorithms [18]. In this study, we have deployed a distributed computing version of such a non-linear motion corrected re-binning reconstruction for free-breathing evaluation of cardiac function. The addition of distributed computing has resulted in acquisition and reconstruction times that are compatible with a clinical workflow and this has enabled a more thorough clinical evaluation of these techniques.

The intent of this study was to explore the clinical applicability of the motion corrected re-binning technique using cloud-based image reconstruction to determine if it can fit into a practical clinical workflow and yield similar cardiac volume measurement results to traditional imaging sequences. The study compared left ventricular end-diastolic volume (EDV), end-systolic volume (ESV), and end-diastolic mass measurements obtained by the retrospective MOtion Corrected re-binning (MOC) imaging sequence, to the clinically standard Breath-Held (BH) and free-breathing AVEraged (AVE) imaging sequences. Measurements were performed by two independent observers to analyze inter-observer reproducibility. Additionally, total combined acquisition and reconstruction times required for the three acquisition techniques were compared.

## Methods

Twenty five healthy volunteers and 25 patients with structurally normal hearts provided written informed consent, and assent when appropriate, to participate in this study. The study was approved by the Institutional Review Board of Children’s National Health System. Sequential subjects who presented for clinical scans or as research volunteers were considered for inclusion in the study without regard to age or clinical status. Volunteers underwent all three scanning techniques; some clinical patients did not undergo AVE imaging due to time constraints associated with performance of their clinical scans and limitations on research scanning time. All images were performed on a single 1.5 T MR scanner (MAGNETOM Aera, Siemens Healthcare, Erlangen, Germany) using a combination of the spine array posteriorly and an 18-channel body matrix array anteriorly. Participants were given breath-hold instruction during scan preparation to assess compliance and were retaught as needed. Image reconstruction of MOC images was performed online using the Gadgetron [17], which was deployed on multiple computational nodes as outlined below.

### Imaging sequences

Our CMR laboratory, which is focused on congenital heart disease work, utilizes standardized imaging protocols based on the subject’s age or size, categorized as infant, child, or teen/adult. These standard sequences were used for acquisition of the ECG-gated 2D segmented BH and AVE images. Typical imaging parameters are shown in Table 1 for all three sequence types. BH sequences in awake patients were acquired per clinical practice using one breath hold per slice and progressing at the subject’s comfort level, with typical imaging time of 12 to 15 s per slice, and a 10 to 15 s rest period between acquisitions. For infants and young children, the BH sequences were performed by utilizing pauses in mechanical ventilation during their clinically-indicated CMR, with similar acquisition and rest times as in awake subjects. Using the lab’s standard imaging protocols, short axis image sequences typically covered the left ventricle from the atrioventricular groove to the apex at end-diastole in 8–10 slices. The exact image slice position and orientation of the initial sequence was copied and pasted for acquisition of subsequent image sequences. For AVE and MOC sequences, subjects were allowed to breathe spontaneously throughout the acquisition, and multiple signal averages (typically 3) were employed for AVE.

**Table 1:** Summary of sequence parameters

	Infant (*n* = 1)		Child (*n* = 6)		Teen/Adult (*n* = 43)	
	BH/AVE	MOC	BH/AVE	MOC	BH/AVE	MOC
FOV (mm^2^)	208 × 208	208 × 208	270 × 270	270 × 270	360 × 360	360 × 360
Matrix	160 × 160	160 × 160	208 × 208	208 × 208	256 × 256	256 × 256
Slice thickness (mm)	6	6	6	6	8	8
Gap (%)	0	0	33	33	25	25
TE (ms)	1.13	1.23	1.13	1.23	1.20	1.19
Echo spacing (ms)	25.3	88.5	25.3	115.4	29.7	135.8
Views per segment	7	30^a^	9	39^a^	11	48^a^
Flip angle (deg)	50	50	50	50	50	50
Acceleration factor	2	4	2	4	2	4

^a^MOC images are re-binned on a higher temporal resolution

Total imaging time for each sequence type was measured from initiation of the sequence on the scanner to display of the completely reconstructed image set on the workstation. Total imaging time includes duration of image acquisition and computation of reconstruction. For BH sequences, this time also includes any rest breaks or time required to reinforce breath-hold instructions with the subject.

### Image reconstruction

Motion corrected image sequences were reconstructed using a distributed Gadgetron implementation [18]. This Gadgetron reconstruction was integrated with the scanner in such a way that the distributed processing was seamless from the user perspective. Images were returned to the scanner immediately after reconstruction without any user interaction. In this study, two different deployment strategies were investigated. One deployment strategy used seven (7) to ten (10) computational nodes deployed in Amazon EC2 (Amazon Web Services, http://aws.amazon.com). These computational nodes were started with a single mouse click from a custom built web interface on the scanner at the start of each patient scan and the nodes were terminated either manually at the end of the patient study or automatically after two hours of idle time. The exact choice of compute nodes and the number of compute nodes varied through the study as new node types became available. A typical choice of compute node was the c4.4xlarge (dual quad-core Intel Xeon E5-2670 and 60GB of RAM) instance type. The total running cost of the computational resources was $6.10–$8.80 per hour. After an upgrade of local computational facilities, a local set of 5 computational nodes was also used. These nodes had 16 cores (four quad-core Intel Xeon CPU E5-2650) and 128GB of RAM. Regardless of the choice of distribution strategy, the reconstruction time remained approximately the same.

### Left ventricular function and mass quantification

All image datasets were de-identified on the scanner, and then subsequently post-processed using commercial QMass software (Medis Medical Imaging Systems, Leiden, Netherlands). Left ventricular endocardial and epicardial borders were traced for measurement of EDV, ESV, and end-diastolic mass using the summation of disks technique, with papillary muscles included in the ventricular volume. Two observers (RC and LO) with 11 and 7 years of cardiac MRI experience independently performed endocardial tracings.

### Statistical analyses

Values of left ventricular volume and mass measurements were determined as mean ± SD for each sequence type and reader. Regression analysis was performed to determine the predictability of the relationship of the FB and AVE acquisition types to the gold-standard BH acquisition type, using the significance level for the F-test at *p* < 0.05 to reject the non-linearity hypothesis. Pearson correlation coefficients were used to test the degree of linear correlation across the various measurement techniques. For each imaging comparison, Bland-Altman plots [19] were constructed to determine the bias and 95 % limits of agreement as ±1.96 SD. Inter-observer variability between the two readers for measurement of EDV, ESV, and mass was tested using the concordance correlation coefficient as described by Lin [20] and McBride [21]. Bland-Altman plots were also constructed to demonstrate inter-observer bias and 95 % limits of agreement. Total scan plus reconstruction times for each acquisition type were determined as mean ± SD, and were tested for significant differences using a paired t-test at the *p* < 0.05 level. Statistical analyses were performed using MedCalc for Windows, version 12.2.1.0 (MedCalc Software, Ostend, Belgium).

## Results

**Fig. 1:**
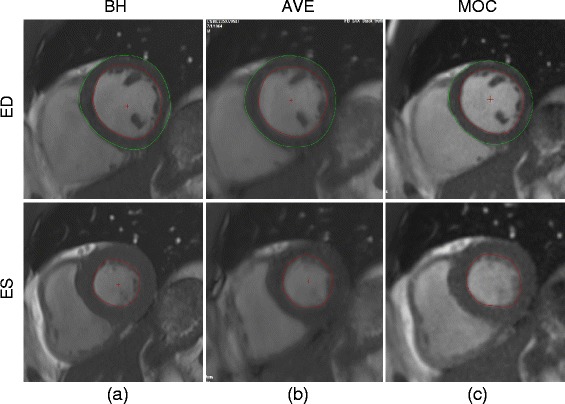
Representative mid-ventricular end-diastolic (ED) and end-systolic (ES) images with and endocardial and epicardial contour tracings for each of three image acquisition types: (**a**) breath-held SSFP, (**b**) free-breathing SSFP, and (**c**) retrospective motion corrected re-binning

Demographics of the study population were (mean ± SD (range)): age, 21.6 ± 11.4 years (2.1–56.6); weight, 57.5 ± 26.9 kg (2.3–101); body surface area, 1.6 ± 0.4 m^2^ (0.4–2.2); 62 % female. Heart rates for each sequence type were: BH 67 ± 11.5 bpm (46–100); AVE 70 ± 10.7 bpm (49–99); MOC 71 ± 11.6 bpm (46–100). BH and MOC sequences were performed in all subjects, while AVE acquisitions were obtained in 11 of the 25 clinical scans due to time constraints specific to the clinical situation and limits on research imaging time. All images were of adequate quality to proceed with volumetric quantification. Representative mid-ventricular end-diastolic and end-systolic image slices for each acquisition type from a single subject with corresponding endocardial and epicardial contours are shown in Fig. 1.

**Fig. 2:**
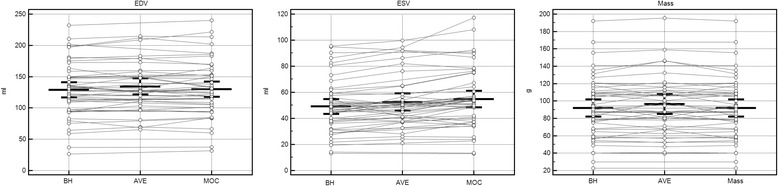
Measured left ventricular end-diastolic volume (EDV), end-systolic volume (ESV), and end-diastolic mass for each of three image acquisition sequences as performed by the primary observer. Bars indicate mean ± 95 % confidence intervals. *BH = breath-held SSFP, AVE = free-breathing SSFP, MOC = retrospective motion corrected re-binning*

**Fig. 3:**
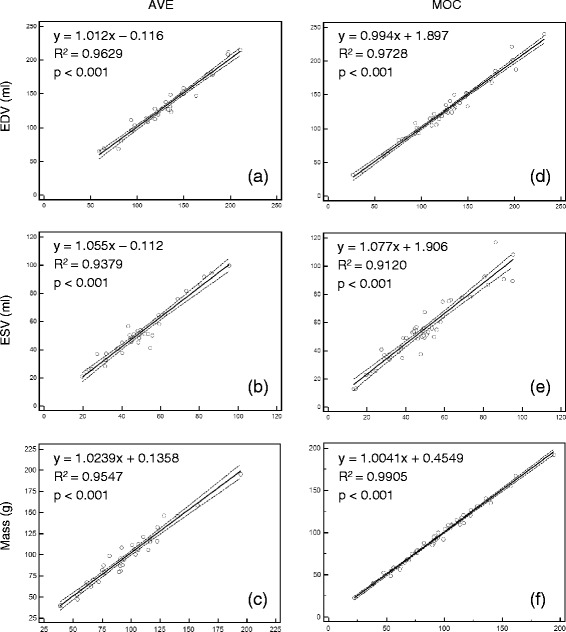
Left ventricular end-diastolic volume (EDV), end-systolic volume (ESV), and end-diastolic mass performed by the primary observer for free-breathing SSFP (**a**, **b**, **c**) and re-binning (**d**, **e**, **f**) acquisitions compared to the clinical gold-standard of breath-held SSFP. The 95 % confidence interval is indicated by the dotted line. *AVE = free-breathing SSFP, MOC = retrospective motion corrected re-binning*

Measured left ventricular EDV, ESV, and mass obtained by the primary observer (RC) for each image acquisition type are shown in Fig. 2. This demonstrates acceptable agreement with regard to the sample means for EDV, ESV, and end-diastolic mass across image acquisition types, although there is a tendency for MOC and AVE to overestimate ESV as compared to BH. Linear regression of the volumetric quantification performed by the primary observer for MOC and AVE acquisition techniques, compared to the gold-standard BH, is shown in Fig. 3. Excellent correlation is demonstrated for MOC and AVE acquisitions compared to BH across a wide range of measurements. The linear relationship is strongest for assessment of EDV and mass, but is also robust for ESV.

**Fig. 4:**
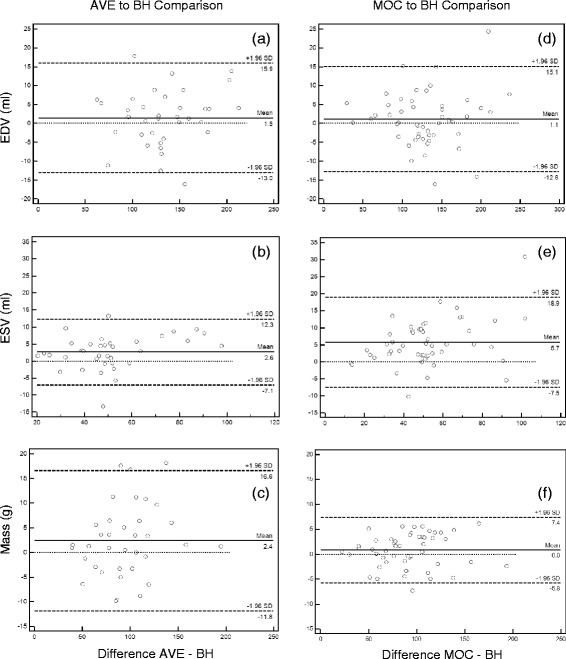
Bland-Altman plots of left ventricular end-diastolic volume (EDV), end-systolic volume (ESV), and end-diastolic mass performed by the primary observer for free-breathing SSFP (**a**, **b**, **c**) and re-binning (**d**, **e**, **f**) acquisitions compared to the clinical gold-standard of breath-held SSFP. *BH = breath-held SSFP, AVE = free-breathing SSFP, MOC = retrospective motion corrected re-binning*

Bland-Altman diagrams in Fig. 4 compare left ventricular volume and mass for MOC and AVE acquisitions to the clinical gold-standard BH performed by the primary observer. Summary of the Bland-Altman statistics as well as Pearson’s correlation coefficient of linearity comparing MOC and AVE techniques to BH are shown in Table 2. The Bland-Altman analysis demonstrates similar performance of the MOC and AVE acquisitions for EDV measurements, with minimal mean bias difference compared to BH (mean bias +1.1. ml for MOC, +1.5 ml for AVE). The performance on measurement of ESV is not as robust as that for EDV, but remains within clinically acceptable parameters (mean bias +5.7 ml for MOC, +2.6 ml for AVE). The measurement of LV end-diastolic mass also compares quite favorably, particularly the MOC technique compared to BH (mean bias +0.8 g for MOC, +2.4 g for AVE). Similarly, the Pearson’s correlation coefficient comparing MOC and AVE EDV measurements to BH is excellent (r > 0.98), while that comparing ESV measurements is slightly less strong (r ≈ 0.95 to 0.96). The correlation between BH and MOC measurement of mass is also quite strong (*r* = 0.9952).

**Table 2:** Summary of Bland-Altman analysis comparing volume and mass measurements by three image acquisition techniques performed by the primary observer

Statistic	EDV (ml)		ESV (ml)		Mass (g)	
	AVE	MOC	AVE	MOC	AVE	MOC
Bland-Altman						
Bias	1.5	1.1	2.6	5.7	2.4	0.8
SD of Bias	7.4	7.1	5.0	6.7	7.3	3.36
Min. Limit (95 %)	−13.0	−12.8	−7.1	−7.5	−11.8	−5.8
Max Limit (95 %)	15.9	15.1	12.3	18.9	16.6	7.4
Pearson correlation coefficient (r)	0.9813	0.9863	0.9685	0.9550	0.9771	0.9952

### Inter-observer reproducibility

**Fig. 5:**
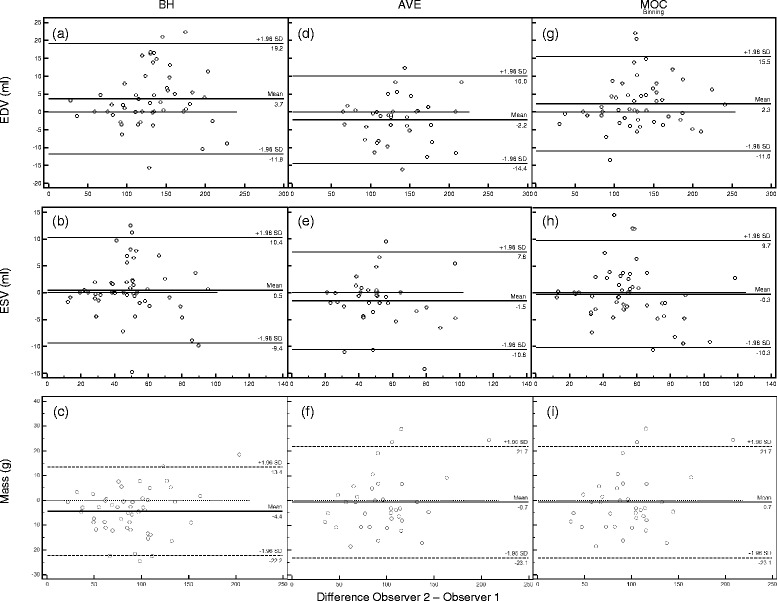
Bland-Altman plots of left ventricular end-diastolic volume (EDV), end-systolic volume (ESV), and end-diastolic mass performed by each of two observers (Obs 1 and Obs 2) respectively for (**a**, **b**, **c**) breath-held SSFP, (**d**, **e**, **f**) free-breathing SSFP, and (**g**, **h**, **i**) retrospective motion corrected re-binning image acquisitions. *BH = breath-held SSFP, AVE = free-breathing SSFP, MOC = retrospective motion corrected re-binning*

Bland-Altman diagrams for the EDV, ESV, and mass measurements performed by the two observers for each image acquisition technique are shown in Fig. 5. Summary of the Bland-Altman statistics and calculated concordance correlation coefficients for inter-observer comparison of EDV, ESV, and mass is shown in Table 3. Overall, minimal mean bias exists between the two observers for measurement of EDV, ESV, and mass across all image acquisition types, and the bias is of the same order of magnitude across measurement groups. The concordance correlation coefficient between observers for EDV and ESV across all acquisition types suggests “substantial strength of agreement” (ρ_c_ 0.95–0.99) based on McBride’s descriptive scale [21]. Using the same criteria, there is substantial strength of agreement for mass measured by the BH and MOC techniques, and mass measured by the AVE technique falls just below this threshold.

**Table 3:** Summary of Bland-Altman and concordance correlation statistics comparing inter-observer variability of volume and mass measurements for three image acquisition techniques

Statistic	EDV (ml)			ESV (ml)			Mass (g)		
	BH	AVE	MOC	BH	AVE	MOC	BH	AVE	MOC
Bland-Altman									
Bias	3.7	−2.2	2.3	0.5	−1.5	−0.3	−4.3	−0.7	−3.1
SD of Bias	7.9	6.2	6.8	5.0	4.6	5.1	9.1	11.4	8.4
Min. Limit (95 %)	−11.8	−14.4	−11.0	−9.4	−10.6	−10.3	−22.2	−23.1	−19.5
Max Limit (95 %)	19.2	10.0	15.5	10.4	7.6	9.7	13.4	21.7	13.2
Concordance correlation coefficient (ρ_c_)	0.9795	0.9852	0.9866	0.9658	0.9678	0.9721	0.9588	0.9486	0.9670
95 % C. I.	0.9645 to 0.9882	0.9712 to 0.9924	0.9768 to 0.9923	0.9409 to 0.9804	0.9384 to 0.9833	0.9520 to 0.9839	0.9299 to 0.9760	0.9050 to 0.9725	0.9428 to 0.9810
Pearson correlation coefficient (ρ)	0.9834	0.9868	0.9882	0.9666	0.9712	0.9734	0.9677	0.9536	0.9711
Bias correction factor (*c*_b_)	0.9961	0.9983	0.9984	0.9992	0.9965	0.9987	0.9909	0.9947	0.9958

### Image acquisition and reconstruction time

**Fig. 6:**
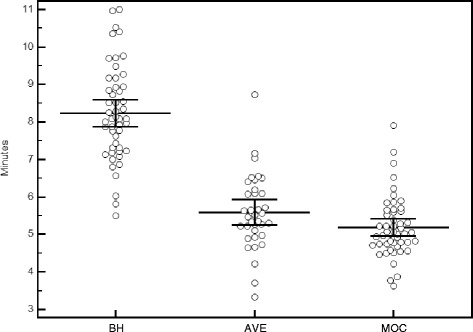
Total image acquisition and reconstruction time in minutes for breath-held SSFP, free-breathing SSFP, and retrospective motion corrected re-binning image sequences. Lines indicate mean ± 95 % confidence intervals. *BH = breath-held SSFP, AVE = free-breathing SSFP, MOC = retrospective motion corrected re-binning*

Total image data acquisition and reconstruction times for each of the three acquisition types are shown in Fig. 6. The mean total acquisition and reconstruction times, as well as comparison amongst each of the three acquisition types are outlined in Table 4. Overall, there is no statistically significant difference in image data acquisition and reconstruction times between the MOC and AVE techniques, but both of these techniques are shorter than standard BH techniques. On average, the MOC acquisition requires 3 min less time than BH imaging, which is a reduction in acquisition time of 37 %. This is similar to the AVE technique, which runs on average 2.5 min shorter than BH.

**Table 4:** Total acquisition and image reconstruction times

Acquisition Time (min)	BH	AVE	MOC
Mean	8.2	5.6	5.2
Range	5.5–11	3.3–8.7	3.6–7.9
SD	1.3	1	0.8
Comparison	BH to AVE	BH to MOC	AVE to MOC
Mean difference	2.5	3	0.2
SD	0.99	1.55	1.09
95 % C. I.	2.209 to 2.881	2.604 to 3.487	−0.167 to 0.568
*p*-value	<0.0001	<0.0001	0.2759

### Age-dependent imaging protocol effect

**Fig. 7:**
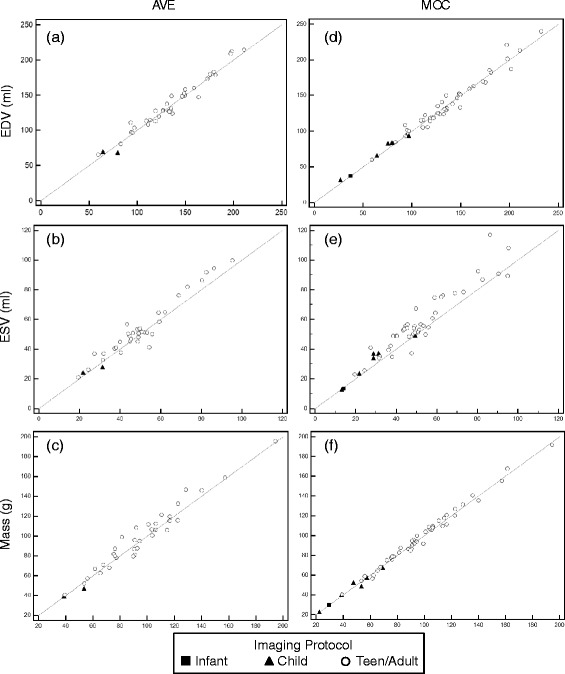
Left ventricular end-diastolic volume (EDV), end-systolic volume (ESV), and end-diastolic mass performed by the primary observer for free-breathing SSFP (**a**, **b**, **c**) and re-binning (**d**, **e**, **f**) acquisitions compared to the clinical gold-standard of breath-held SSFP, with age-dependent imaging protocols indicated. *AVE = free-breathing SSFP, MOC = retrospective motion corrected re-binning*

**Fig. 8:**
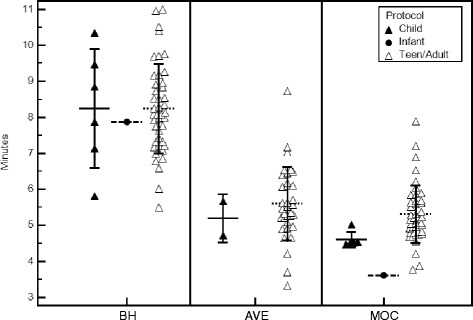
Total image acquisition and reconstruction time in minutes for the three image acquisition techniques subdivided into age-dependent imaging protocol groups. Lines indicate mean ± 1 SD. *BH = breath-held SSFP, AVE = free-breathing SSFP, MOC = retrospective motion corrected re-binning*

Due to the clinical nature of this study, only 7 subjects (14 %) had imaging performed outside of the teen/adult protocol parameters (infant = 1, child = 6). This small sample size limited the ability to perform meaningful statistical analysis on the age-dependent protocol differences; however, some information can be gleaned. Figure 7 demonstrates the same linear regression analysis as presented in Fig. 3, but in addition indicates the imaging protocol used for each subject. The subjects who had imaging performed using the infant and child imaging protocols cluster along the line of unity, suggesting that the imaging protocol parameters likely have no substantial effect on results. Similarly, total image acquisition and reconstruction times for each acquisition type subdivided into the age-dependent imaging protocol used are shown in Fig. 8. Total image acquisition and reconstruction times for each protocol (with the exception of the single infant for MOC) tend to cluster around similar means, suggesting that the age-dependent protocol used does not substantially affect the results within an acquisition type. While these analyses suggest that the variations in protocol parameters used for various age groups do not substantially affect the volume, mass, or acquisition time results, additional studies in these younger age populations would be required to create meaningful statistical outcomes.

## Discussion

The purpose of this study was to investigate the ability of the motion corrected re-binning image reconstruction technique to provide reliable functional measurements, and to explore workflow feasibility through the use of cloud-based image reconstruction. Analysis of the results demonstrates that the MOC technique compares favorably to the gold standard BH technique for measurement of left ventricular EDV, ESV, and end-diastolic mass, with the correlation being strongest for EDV and mass. A recent multicenter analysis of expert readers at 7 preeminent adult cardiac MRI centers demonstrated the following bias results when measuring left ventricular volumes and mass on 15 identical image data sets: EDV −36.6 to 40.5 mL (average 0.9 mL); ESV −32.9 to 41.2 mL (average 0.8 mL); mass −44.5 to 59.6 g (average 0.7 g) [22]. If one considers these types of bias results across expert CMR readers as a measure of the current clinical standard, then the bias between MOC and AVE results compared to BH found in our analysis are certainly well within the range of clinical acceptability. There is a tendency for MOC to overestimate the ESV compared to the BH technique. This tendency is similar to that seen in AVE acquisitions, and was present in both independent observers. We hypothesize that this is likely due to endocardial blurring resulting from in-plane motion that is exaggerated by signal averaging in both techniques. Analysis demonstrates that the observed bias is small, and in most cases likely not clinically significant, but should be taken into consideration when utilizing either of the free-breathing techniques. Inter-observer reproducibility of the MOC imaging technique is also shown to be similar to that of traditional breath-held SSFP volumetric imaging.

The primary advantage of the MOC technique compared to the traditional BH technique is that images can be reliably obtained in a free-breathing patient in a fraction of the time needed for breath-holding, and yield ESV and EDV results that are comparable and clinically valuable. For AVE imaging, it should be noted that the total imaging time reported is primarily devoted to data acquisition with a very small amount of time devoted to image reconstruction, so the total imaging time reported for AVE cannot be substantially shortened. Conversely, the total imaging time reported for MOC imaging includes data acquisition as well as reconstruction time at the end. With further optimization of the reconstruction algorithm, the MOC acquisition could potentially be shortened without reducing image quality. This would increase the favorability of MOC imaging over AVE imaging by reducing the imaging time even further. Also, the ability to perform MOC image reconstruction in the cloud allows for continued simultaneous acquisition of other images on the scanner while the MOC scan is undergoing reconstruction, which can have the effect of shortening the overall study time and improving clinical workflow.

As seen in Table 4, the standard deviation of imaging time is greatest for BH imaging and least for MOC imaging. This implies that the time required to acquire BH images has a greater variability in practical clinical workflow, with dependence on a number of factors including subject compliance, comprehension of breath-hold instructions, need for anesthesiologist to perform mechanical breath holds, etc. The acquisition time for AVE and MOC images is dependent primarily on the subject’s heart rate, optimization of the number of segments for AVE, and the time allocated for image reconstruction. Therefore, use of the MOC acquisition technique can potentially lead to a more predictable workflow, while providing robust and efficient cine imaging. Although not analyzed in this study, the images obtained by the MOC technique are subjectively of higher quality with less endocardial border blurring compared to AVE imaging (see Fig. 1), and tend to be more reliably produced, thus obviating the need to repeat unsatisfactory image slices that oftentimes occur when attempting to use AVE imaging in clinical analysis. Further study is necessary to appropriately compare the robustness and image quality of the MOC sequence to that of BH or AVE.

In this study we have not reported actual reconstruction times for the MOC scans since they would likely be somewhat misleading. Specifically, if a full dataset from a MOC scan is reconstructed after the scan on cloud computing resources, the reconstruction time is fairly short since multiple slices are reconstructed in parallel, i.e., the reconstruction time will be on the order of a couple of minutes. In a real acquisition, it is not possible to start reconstruction of the final slice until the data is actually acquired and consequently the time limiting step is actually how long it takes to reconstruct data from one slice, which is on the order of one minute per slice [12]. The images are usually reconstructed within a minute after the end of the acquisition.

This study uses two different kinds of distributed computing strategies. One is based on rented cloud computing resources from a commercial vendor and one is based on locally deployed servers. Both strategies provide the same performance in terms of reconstruction time, but there are other trade-offs to consider. The locally deployed hardware has a high up-front cost to purchase the hardware, it scales relatively poorly when new scanners are added, and there is also cost associated with housing and maintaining local hardware. However, with locally deployed hardware the resources are always on and there is no need to start or stop the resources, which makes it more transparent to the user. The rented resources, on the other hand, have no up-front cost and can be deployed on demand as more systems and users are added. The cost of running the commercial cloud resources (on the order of $10 per hour) is insignificant in the context of scanning a patient, but there are some issues to consider when connecting a clinical system directly to external cloud resources. First, the clinical system must be equipped with a 1GB/s Internet connection to accommodate the data as it is acquired. Second, care must be taken to ensure that no patient identifiers are transferred to the cloud computing resources. This is easily achieved, since the computational resources do not need to know the patient identity or other identifiable metadata. A further level of safety can also be added by using encrypted tunnels to connect to the cloud resources (as was done in this study). Finally, the staff operating the scanner must be comfortable starting and stopping external resources to avoid accruing excessive costs associated with idle resources. The preferred strategy will likely vary from site-to-site depending on existing IT infrastructure and available Internet connection speeds. Nonetheless, the MOC cloud-based image reconstruction technique can fit easily into most clinical workflows, with results available directly from the scanner.

### Study limitations

This study intentionally included clinical patients so as to include a wide range of subject size and imaging parameter variations, as is typically seen in a pediatric CMR laboratory. However, this resulted in a subset of subjects in whom FB acquisitions could not be performed due to constraints on imaging time, making comparisons to the FB group less robust. Likewise, the limited number of subjects imaged outside of the teen/adult protocol parameter group limits the ability to perform meaningful statistical analysis of this variation. It is also acknowledged, that while the results of volume and mass measurements performed by the MOC technique compare favorably to those obtained by the gold-standard BH technique, care should be taken before using volumetric data from MOC images to make prognostic determinations in clinical algorithms that are based on SSFP imaging.

## Conclusion

Motion corrected re-binning image reconstruction provides robust cardiac imaging that can be used for ventricular volume quantification. Images compare favorably to traditional breath-held SSFP imaging, and can be obtained in a fraction of the time when using cloud-based distributed computing image reconstruction.

## Abbreviations

AVE: free-breathing steady state free-precession imaging with multiple image averaging
BH: breath-held steady-state free precession imaging
Bpm: beats per minute
CMR: cardiovascular magnetic resonance
EDV: end-diastolic volume
ESV: end systolic volume
MOC: motion corrected re-binning real time imaging sequence
SSFP: steady-state free precession
